# Neural kernels for recursive support vector regression as a model for episodic memory

**DOI:** 10.1007/s00422-022-00926-9

**Published:** 2022-03-29

**Authors:** Christian Leibold

**Affiliations:** Fakultät für Biologie & Bernstein Center Freiburg, Albert-Ludwigs-Universität Freiburg, Hansastr. 9a, Freiburg, 79104 Germany

**Keywords:** Theta sequences, Episodic memory, Recursive support vector regression

## Abstract

Retrieval of episodic memories requires intrinsic reactivation of neuronal activity patterns. The content of the memories is thereby assumed to be stored in synaptic connections. This paper proposes a theory in which these are the synaptic connections that specifically convey the temporal order information contained in the sequences of a neuronal reservoir to the sensory-motor cortical areas that give rise to the subjective impression of retrieval of sensory motor events. The theory is based on a novel recursive version of support vector regression that allows for efficient continuous learning that is only limited by the representational capacity of the reservoir. The paper argues that hippocampal theta sequences are a potential neural substrate underlying this reservoir. The theory is consistent with confabulations and post hoc alterations of existing memories.

**Supplementary Information:**

The online version contains supplementary material available at 10.1007/s00422-022-00926-9.

## Introduction

To retrieve episodic memories, brains need to elicit robust internal sequences of neuronal activity patterns that are linked to previous sensory-motor experiences. Thus, neural processes need to be in place that form such activity sequences as well as link them to sensory-motor areas while learning. Episodic memories are further known to be able to change over time by reconsolidation (Sara 2000; Nader et al 2000; Milekic and Alberini 2002; Alberini and Ledoux 2013), eventually even leading to false memories of events that never happened (Loftus 1992; Hyman Jr. et al 1995). This suggests that the architecture of episodic memory is versatile and local in time in the sense that any pair of memory items can be connected into a memory episode independent of context.

Since electrophysiological recordings in animals prohibit correlating activity sequences to introspective retrieval of episodic memories, memory-related activity sequences are typically studied in rodents in association with behavioral performance in navigational tasks (Lee and Wilson 2002; Karlsson and Frank 2009). Activity sequences of hippocampal place cells thereby have been reported to correlate to (Lee and Wilson 2002; Dragoi and Buzsáki 2006; Foster and Wilson 2007) and to causally explain (Jadhav et al 2012; Fernández-Ruiz et al 2019) memory-dependent navigation. Sequences have furthermore been found to exist even before a specific spatial experience has been made by an animal (Dragoi and Tonegawa 2011, 2014; Farooq and Dragoi 2019), suggesting that, at least part of the learning process is about establishing synaptic connections between existing intrinsic neuronal sequences and the sensory-motor areas that represent the content of the memory episode.

**Fig. 1:**
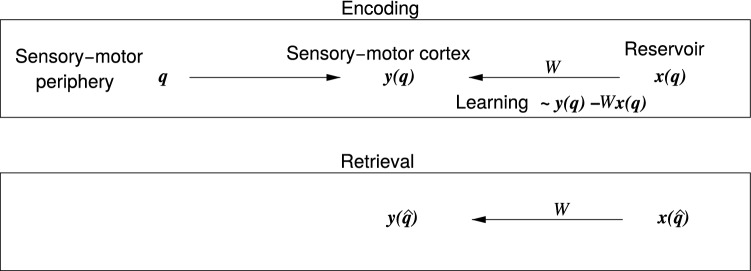
Conceptual overview. At any instance in time, let us consider $\varvec{q}$ to encode any sensory-motor experience of an agent (human, animal or machine). A neocortical representation $\varvec{y}(\varvec{q})$ of this experience is evoked by the sensory afferents, and motor efference copies. The current state $\varvec{x}$ of a reservoir (e.g., in the hippocampal formation) is linked to the temporally coincident experience $\varvec{q}$ by synaptic learning of the connections *W* from the reservoir to the neocortex. The synaptic change is thereby proportional to the error signal $\varvec{y} - W\varvec{x}$; see Eq. (5). During retrieval, the reservoir state $\varvec{x}$ previously associated with a real, or confabulated experience $\varvec{{\hat{q}}}$, evokes a corresponding neocortical representation $y(\hat{q})$

The idea that multi-purpose intrinsic neuronal dynamics is used to represent time series of extrinsic events has been invented multiple times under the names of echo-state networks (Jaeger and Haas 2004), liquid computing (Maass et al 2002) and reservoir computing (Jaeger 2005; Schrauwen et al 2007; Lukoševičius and Jaeger 2009) and has proven to be both computationally powerful and versatile (Maass et al 2002; Sussillo and Abbott 2009), particularly, since multiple output functions can be learned on the same intrinsic activity trajectory (sequence) and played out in parallel.

There has been considerable previous work on how to construct a dynamical reservoir via the dynamics of neuronal networks (Haeusler and Maass 2007; Sussillo and Abbott 2009; Lazar et al 2009). Also different learning rules for the synapses from the sequence reservoir to the output neurons were successfully explored, such as the perceptron rule (Maass et al 2002), a Hebb rule (Leibold 2020) or recursive least squares-derived rules (Williams and Zipser 1989; Stanley 2001; Jaeger and Haas 2004; Sussillo and Abbott 2009). The more general applicability of reservoir computing to neuroscience is, however, still limited because several open questions remained, particularly about how to relate reservoir computing ideas to neurophysiological data: For example, can sufficiently rich reservoirs be realized with spiking neuronal networks? How can be found out whether reservoir spiking activity bears meaningful representations in the sense of Marr’s second level—as opposed to just being a “liquid” black box? Can a regression type learning rule be neuronally implemented using local Hebbian principles? How can new information (including false memories) be added to an existing episode or specific memory items be deleted? Can physiologically plausible models realize the universal approximation property (Grigoryeva and Ortega 2018) or are the limits of learning already imposed by interference of weight updates at output synapses below the capacity limit (Amit and Fusi 1994)? Particularly the latter problem is of fundamental importance in applying reservoir computing ideas to brain activity data, since available recordings (and also most models) are generally restricted to only a relatively small number of neurons (limiting representational capacity), whereas a whole real brain has been close to infinite capacity for all practical (here experimental) purposes. Finding a representation of reservoir activity would thus eliminate capacity limitations and allow for efficient representation of a huge set of sensory-motor experiences in the synaptic weights.

Here, I propose a neuronal implementation of recursive kernel support vector regression as an *efficient one-shot* learning rule that is only limited by the representational capacity of the dynamical reservoir and allows for importance scaling (as compared to only graceful decay). Kernels thereby allow reservoir activity to be interpreted as representations in the sense of Marr (Hermans and Schrauwen 2012), which adds to theoretical neuroscience by allowing for specific interpretations of neural activity. For example, I will argue that theta sequences of hippocampal place cells (Foster and Wilson 2007) implement a kernel that represents distance in time or space and that the integration of auditory nerve activity at different delays implements a kernel representing time for acoustic stimuli in a cochlear frequency band. Below the capacity limit, the learning rule implements the well-known recursive (Gauss-Legendre) least mean squares (or FORCE) rule (Sussillo and Abbott 2009) on the underlying neuronal patterns, showing that FORCE-learning is only limited by the capacity of the simulated or measured reservoir.

## Results

Let us consider an episodic experience to be fully reflected by the sensory-motor evoked summed postsynaptic potentials $y^{(k)}_t$ at all involved neurons *k* for all points in time *t*. In order to store the episodic experience as a memory, the synaptic inputs $y^{(k)}_t$ need to be linked to a preexisting reservoir state $\varvec{x}_t$ such that, whenever $\varvec{x}_t$ is present afterward, the learned synaptic connections $\varvec{w}^{(k)}$ from the reservoir evoke the same depolarizations

1 $$\begin{aligned} y^{(k)}_t = \varvec{x}_t^\mathrm{T}\, \varvec{w}^{(k)} \end{aligned}$$

without the presence of the original sensory-motor activity (Fig. 1), i.e., $\varvec{w}^{(k)}$ solve a regression problem with $\varvec{x}_t$ as regressors. Considering only depolarizations $y^{(k)}$ in such a one-layer feedforward network, one does not need to consider nonlinearities during spike generation and, with reasonable approximation, the model is an effectively linear network. It also should be noted that in this paper I do not intend to explain the nature of the preexisting sequences $\varvec{x}_t$, and just assume that they exist. For the sake of simplicity, I further drop the neuron index *k*, since all considerations trivially generalize to multiple neurons.

Besides the scalar product in Eq. (1), biological feasibility imposes two more constraints on how one models learning. First, synaptic plasticity should be activity-dependent and therefore the weights should be a superposition of existing neuronal activity patterns,

2 $$\begin{aligned} \varvec{w} = \sum _{t=1}^P \varvec{x}_t\, u_t = X\, \varvec{u} \end{aligned}$$

with $X=(\varvec{x}_1,\dots ,\varvec{x}_P)$ (see Sect. 4 on representer theorem). Second, the learning rule needs to be recursive, i.e., new input–output pairs $(\varvec{x}_{P+1},y_{P+1})$ should be added such that Eq. (1) holds for all previous patterns (no interference) until the capacity limit and memory decay beyond the capacity limit should be importance based. In short, the learning rule is supposed to identify the loads $\varvec{u}$ such that the outputs $y_t$ are exactly recovered by the model,

3 $$\begin{aligned} \varvec{y} = X^\mathrm{T}X\, \varvec{u}\ . \end{aligned}$$

As long as the kernel matrix $K=X^\mathrm{T}\, X$ is invertible (below the capacity limit), the solution for $\varvec{u}$ is exact and straightforward. For non-invertible or badly conditioned *K* (at or above the capacity limit), the standard approach would be to use the pseudo-inverse $K^*$ of *K*, which optimizes the mean squared deviation between output $\varvec{y}$ and model output $K\, K^*\, \varvec{y}$ and leads to the classical recursive least squares (RLS) algorithm if applied recursively. RLS on the loads $\varvec{u}$, however, has two main disadvantages. First, RLS makes explicit use of time making it hard to modify memories by post hoc insertion of new detail within an existing memory sequence. Second, RLS on the loads $\varvec{u}$ is hard to interpret biologically.

I therefore suggest, as an alternative approach, to solve the regression problem by maximizing

4 $$\begin{aligned} {{\mathcal {W}}}(\varvec{u}) = -\frac{1}{2}\, \varvec{u}^\mathrm{T}\, K\, \varvec{u} + \varvec{y}^\mathrm{T}\, \varvec{u}\ , \end{aligned}$$

which, for invertible *K*, yields the exact recovery condition Eq. (3), therefore justifying the use of ${{\mathcal {W}}}$ as the underlying objective function. Moreover, the maximization problem from Eq. (4) can be derived as the dual problem of support vector regression for $\varepsilon$-insensitive loss (see Sect. 3 and Vapnik 1995; Schölkopf and Smola 2002), further supporting the interpretation of regression.

Since support vector approaches translate to nonlinear models using the kernel trick $K_{nm}=\varvec{x}_n^\mathrm{T}\varvec{x}_m \rightarrow \kappa (n,m)$ (Vapnik 1995), the model also provides a foundation for neural implementations of kernels, which can be considered as *representations* of the topological space spanned by *n* and *m*. In the same sense as Marr saw representations to be connected to the algorithmic level, the kernel represents the space of *n* and *m* in a sufficient way to fully specify the outlined regression algorithm, and thus, following (Hermans and Schrauwen 2012), I suggest to consider it as being the true neural representation of this space in contrast to considering representations as activity patterns in undersampled cell populations.

Maximizing ${{\mathcal {W}}}$ results in an update rule for $\varvec{u}$ (see Sect. 3) that translates into a weight change $\varvec{\Delta w} = X\varvec{\Delta u}$ of

5 $$\begin{aligned} \varvec{\Delta w} = \underbrace{(y_P - \varvec{w}^\mathrm{T}\,\varvec{x}_P)}_{e_P}\,\frac{{{\mathcal {N}}} \varvec{x}_P}{\varvec{x}_P^\mathrm{T}\, {{\mathcal {N}}}\, \varvec{x}_P} \ . \end{aligned}$$

with ${{\mathcal {N}}}=1\!\mathrm{l}-X\, K^{-1}\, X^\mathrm{T}$, and an iteration rule

6 $$\begin{aligned} {{\mathcal {N}}} \leftarrow {{\mathcal {N}}} - \frac{ ({{\mathcal {N}}}\varvec{x}_P)\, (\mathcal{N}\varvec{x}_P)^\mathrm{T}}{\varvec{x}_P^\mathrm{T}{{\mathcal {N}}}\varvec{x}_P}\ , \end{aligned}$$

that is equivalent to RLS without forgetting (i.e., without regularization). The learning rule is one shot in the sense that, for any new pattern, the update rules have to be applied only once and it allows for the functional interpretation *error* ($e_P$) times *novelty* (${{\mathcal {N}}}$): Because $1\!\mathrm{l}-{{\mathcal {N}}}$ is a projection operator (see Sect. 3), ${{\mathcal {N}}}\varvec{x}_P$ will be 0 whenever ${\varvec{x}}_P$ equals one of the previous patterns already included in *X*, whereas any component of $\varvec{x}_P$ that is orthogonal to all patterns in *X* will be unaffected by ${{\mathcal {N}}}$. The action of ${{\mathcal {N}}}$ can thus be computationally interpreted as *novelty detection*. For a naive learner ($P=0$), the rule is plain Hebbian, since the error equals the output and the novelty equals the input pattern. In Sect. 4, I will suggest a biologically feasible implementation of ${{\mathcal {N}}}$ and its learning as anti-Hebbian updates of a recurrent neural network. Importantly, the translation into neuron space resulting in Eqs. (5) and (6) is only required to show how the learning rule can be biologically implemented. In contrast to RLS, it is not necessary to use these update rules for all ensuing applications, which are only relying on the numerically much more tractable update rule for the loads ${\varvec{u}}$ presented in Eq. (7) in Sect. 3.

**Fig. 2:**
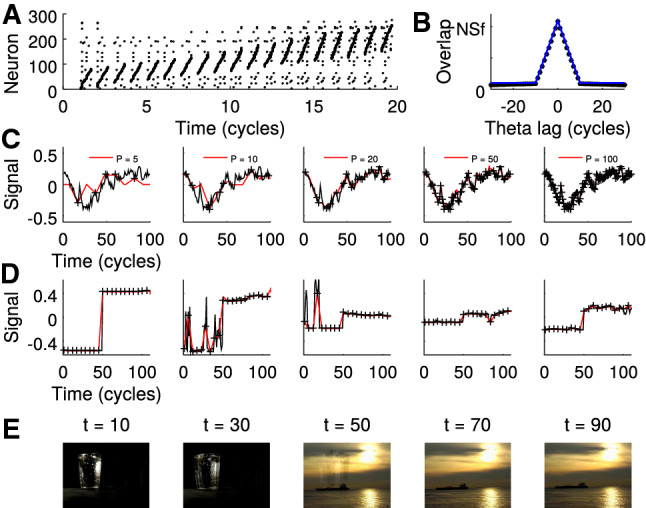
Episodic learning with theta sequences. (A) Spike raster plot of the first 300 of $N=10,000$ neurons implementing theta sequences as described in Sect. 3 *Theta sequences* (sparseness $f=0.01$, sequence length $S=10$). In every theta cycle the sequence moves one neuron upward. (B) Kernel derived as scalar product between population patterns from the simulations shown in A (black dots) and theoretical prediction (blue line). (C) Retrieval (red line) of a low-pass noise signal (black) of length $T=100$ from *P* observations (crosses; for *P* see insets from left to right) using the theoretical kernel from B. The signal was generated as a running average (50 time steps) of white noise. (D) Same as B. Brightness signal for five example RGB channels from a movie scene ($P=20$, $T=111$, $N=576\times 768 \times 3$). (E) Retrieval of movie snippet (five example frames shown) from *.re_potemkin*, a copyleft crowd sourcing free/open source cinema project (https://re-potemkin.httpdot.net/). Original movie snippet and reconstruction are provided as Videos S1 and S2

As a first neuroscience application, I refer to hippocampal theta sequences (Fig 2A): Roughly, one considers a subset of place cells to fire in sequence in every cycle of the hippocampal theta oscillation of the local field potential (about 8 Hz in rodents). In the subsequent cycle, the starting neuron of the previous cycle drops out of the sequence but a new neuron is added at the end of the sequence. Thus the activity patterns of close-by cycles are similar, whereas they become more and more distinct the further the cycles are spaced apart.

In the simple theta sequence model outlined above, the overlap (scalar product) of activity patterns decays linearly (see Sect. 3) implementing a kernel $K_{mn}=\kappa (n-m)$ as a function of the distance $n-m$ of the two cycles (Fig. 2B).

Inserting the triangular linear kernel from Fig. 2B into the learning rule derived by recursively maximizing ${{\mathcal {W}}}$, one can recover the original signal $y_t$ without simulating the underlying reservoir. Increasing detail of the original signal can be retrieved the more pairs ($\varvec{x}_t, y_t$) one takes into account for learning (Fig. 2C). Since the kernel is a continuous function, the capacity has become infinite, i.e., any function $y_t$ can be recovered if the neuron number *N* becomes infinite.

As mentioned above, generalization to multiple neurons is trivial, and to illustrate let us consider each output neuron to reflect one RGB color channel of any pixel in a movie ( 1.3 million neurons). Using only 20 of 110 movie frames already allow for recovery of the movie snippet with a compression below 20% (Fig. 2D, E).

**Fig. 3:**
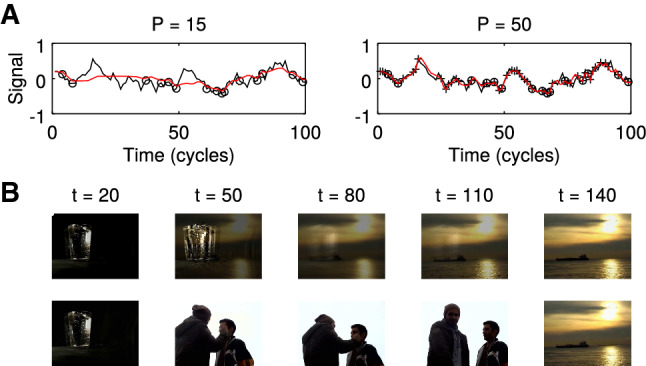
Post hoc addition of memory items. **A** Left: Retrieval (red) of a low-pass noise signal (see Fig. 2C) of length $T=100$ for $P=15$ randomly positioned inputs (circles). Right: Same as left after 35 further inputs (crosses) have been iteratively added to the learning process. **B** Illustration of A for post hoc insertion of a movie scene. Top: original movie sequence ($P=20$). Bottom: Movie sequence after a new scene has been inserted to the original snippet ($P=35$). Movies are provided in Videos S3 and S4

By construction, the learning rule has no explicit dependence on time; thus, the order in which pairs $(\varvec{x}_t,y_t)$ are presented makes no difference to the final fit (Fig. 3A), which is not the case for the FORCE rule derived from classical least squares.

Biologically, this means that any episode can be post hoc modified by learning new pairs $(\varvec{x}_t,y_t)$ with temporal contingencies reflected in the kernel arguments, generating a model of false memories (Fig. 3B).

**Fig. 4:**
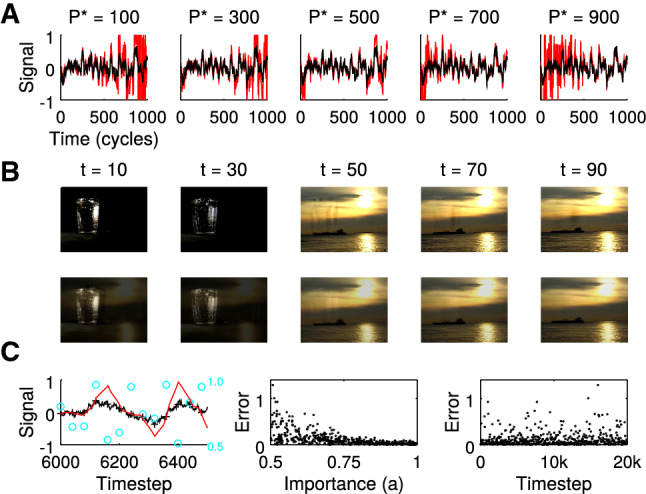
Importance scaling. **A** Retrieval (red) of a low-pass noise signal (black; see Fig. 2C) for attenuation parameters $a_t=\lambda ^{\vert P^*-t\vert }$ with $\lambda =0.999$ and varying importance centers $P^*$ (see titles). **B** Illustration using the movie snippet from Fig. 2 with importance in the beginning ($a_t=\lambda ^{t}$,top) and in the end ($a_t=\lambda ^{110-t}$, bottom). In the image sequence on top one stills sees an erroneous reflection of the glass in the last three images, whereas in the bottom sequence the glass in the first to frames shown erroneously displays the yellowish colors from the end. Movies are provided in Videos S5 and S6. **C** Left: Retrieval (red) of a low-pass noise signal (black) with $N=20,000$ time steps (only shown between time step 6,000 and 6,500) and $P=500$ patterns (crosses) with random importance values *a* (cyan) between 0.5 and 1. Middle: Reconstruction error (absolute difference between black an red line) negatively correlates with *a* for all $P=500$ patterns. Right: Error has no dependence on time

Every memory system is finite and the way of forgetting fundamentally determines its usefulness for practical applications. A graceful decay of memories over time (Amit and Fusi 1994) is already quite an advantage to catastrophic forgetting in attractor networks (Hopfield 1982); however, the behavioral relevance of a memory may not just depend on how old or young it is. I therefore introduce an importance scaling into the learning rule in that loads $u_t$ are multiplied with some attenuation factor $0\le a_t\le 1$. Thus, if one chooses $a_t = \lambda ^{(T-t)}, 0<\lambda <1$ one retains a graceful decay over time as in standard RLS. The resulting learning rule that maximizes the modified ${{\mathcal {W}}}$ is then obtained by only the small modification of replacing the kernel $\kappa (n,m)$ by $\kappa (n,m)\, a_n\, a_m$ (see Sect. 3). The effect of importance scaling is illustrated in Fig. 4A, B, where the learning rule is told to pay more attention to a certain time interval at the cost of worse reconstruction in other time intervals.

**Fig. 5:**
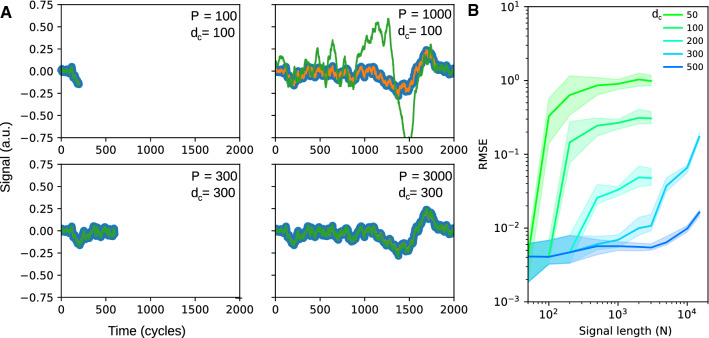
Capacity. **A** Example reconstructions (green) of a signal (orange) for smaller (top) and larger (bottom) cutoff dimensions ($d_c=100$ and 300, respectively). Sample points used for reconstruction (signal length *p* = cycles / 2) are shown in blue. For the left panels the signal length equals the cutoff dimension. On the right the signal is 10 times longer than the cutoff (only 1000 data points shown). **B** Reconstruction error (root mean squared) for different cutoff dimensions (colors as indicated) as a function of signal length (solid lines indicate mean from 20 repetitions, shaded areas the 90 percent quantile). The results are derived from a low-pass noise signal with a running average over 100 time steps and a triangular kernel with length 25 time steps

Importance may randomly vary over time and thus temporal contingency in *a* values should not be a necessary prerequisite for importance scaling. Applying the learning rule in a scenario with random *a* values shows that retrieval error is indeed largest for small *a* independent of time (Fig. 4C). Post hoc increase of *a* could thus be considered as a model of memory consolidation, post hoc decrease of *a* as a model of extinction learning.

With importance scaling as a weighting mechanism at hand, let us now revisit the original capacity question. In the language of the recursive updating rules from equations (7) and (8) the memory and computational demand scale with square of the number of patterns *P*. A straightforward choice to limit the capacity is to introduce a cutoff dimension $d_c$ such that only the $d_c$ patterns with highest importance values *a* are stored in the algorithm and the other dimensions are set to 0. In Fig. 5A, B I vary $d_c$ for low-pass filtered noise signals of different length with linearly increasing importance toward the signal end and observe that for low $d_c$, the reconstruction error increases relatively soon, whereas for $d_c \gtrapprox 300$ reconstruction worked well even for signal lengths up to 10 times larger than $d_c$, which reflects that the geometry of the kernel fits the correlational structure of the signal.

**Fig. 6:**
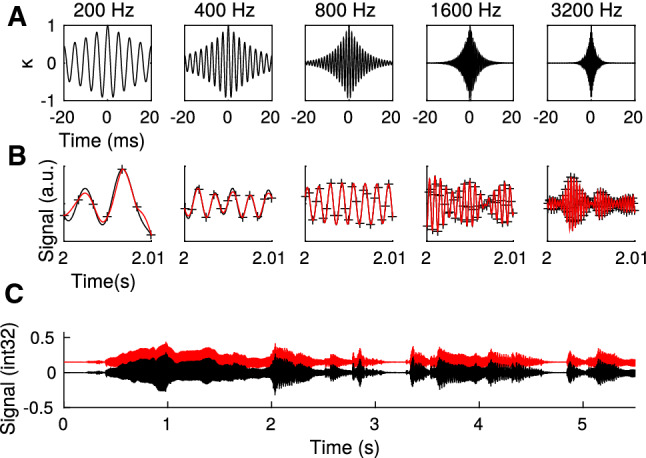
Sound reconstruction. **A** Kernels representing time in a frequency channel with center frequency on top (see Sect. 3 *Frequency kernels*). **B** Retrieval (red) of the signal (black) in five of the frequency channels (crosses mark memory items). **C** Reconstruction (red; moved upward for reasons of illustration) of the original sound signal (black; the beginning of the song http://ccmixter.org/files/texasradiofish/63300, CC BY NC) by summing over the filter-weighted channel components (see Sect. 3 *Frequency kernels*). Reconstructed sound file is provided in Audiofile S8, well as the identically filtered original sound wave (Audiofile S7)

The need to adjust the kernel length to the time scale of signal fluctuations suggests that more specific signal properties require more specifically designed kernels. In most neuroscience applications, sensory signals are not random but reflect physical constraints of the environment or the sensory periphery. As a next example I therefore consider functions with bandpass characteristics similar to cochlear frequency channels. Knowledge about the preferred local structure of a function (oscillations with a certain frequency) suggests a kernel with similar bandpass characteristics (see Sect. 3 and Fig. 6A). In contrast to the triangular linear kernel which only represents temporal distance, the band kernels represent temporal distance (by their decay) and frequency.

Learning is then performed on each cochlear frequency channel separately and the fitting benefits from both recovering the function values at a few points and the fine structure of the kernel. A post hoc synthesis across frequency channels recovers the original soundwave with high fidelity and smaller memory demand as the original sampling (see Sect. 3 *Frequency kernels*).

## Methods

### Recursive support vector regression

Linear support vector regression with $\varepsilon$-insensitive loss (Schölkopf and Smola 2002; Vapnik 1995) is derived from minimizing the squared L$_2$-norm $\frac{1}{2}\Vert \varvec{w}\Vert ^2$ of the weight vector of the linear model $f(x)=\varvec{w}^\mathrm{T}\, \varvec{x} + b$ under the inequality constraints $-(\varepsilon + \zeta _n) \le y_n - f(\varvec{x}_n) \le \varepsilon + \zeta _n^*$, with $\zeta _n,\zeta _n^*\ge 0$, and including the sum of slack variables $\sum _n(\zeta _n + \zeta ^*_n)$ as a regularizer.

The classical work has shown that the resulting optimal solution yields a weight vector of shape

$$\begin{aligned} \varvec{w} = \sum _n (\alpha _n^*-\alpha _n)\, \varvec{x}_n \end{aligned}$$

that maximizes the dual problem

$$\begin{aligned} {{\mathcal {W}}}(\varvec{u},\varvec{v}) = -\frac{1}{2}\, \varvec{u}^\mathrm{T}\, K\, \varvec{u} + \varvec{y}\varvec{u} - \varepsilon \sum _n v_n \end{aligned}$$

with $K_{nm}=\varvec{x}_n^\mathrm{T}\, \varvec{x}_m$, $u_n=\alpha ^*_n - \alpha _n$, $v_n = (\alpha ^*_n+\alpha _n)$ under the constraints $\alpha ,\alpha ^*\ge 0$. Hence, for every local maximum of ${{\mathcal {W}}}$ regarding $\varvec{u}$, there is a combination of $\alpha _n,\alpha _n^*$ that minimizes $\sum _n v_n$, i.e., $\alpha _n=0$ if $u_n>0$ and $\alpha _n^*=0$ if $u_n<0$. For $\varepsilon \rightarrow 0$, the maximum in $(\varvec{u},\varvec{v})$ converges to $\alpha _n=0$ or $\alpha _n^*=0$, and thus, in this limit, one can drop $\varvec{v}$ from the equations.

Here, a recursive learning rule is derived such that $\mathcal{W}$ remains at this maximum if a new observation $(y_p,\varvec{x}_P)$ is added. One therefore denotes $\varvec{u}^\mathrm{T} = (\varvec{{\tilde{u}}}^\mathrm{T}, u_P)$, $\varvec{y}^\mathrm{T} = (\varvec{{\tilde{y}}}^\mathrm{T},y_P)$, and ${\tilde{X}} = (\varvec{x}_1,\dots ,\varvec{x}_{P-1})$ and finds the optimum of

$$\begin{aligned} {{\mathcal {W}}}\left( (\varvec{{\tilde{u}}}^\mathrm{T},u_P)^\mathrm{T}\right)= & {} -\frac{1}{2}\, \varvec{{\tilde{u}}}^\mathrm{T}\, {\tilde{K}}\, \varvec{{\tilde{u}}} - u_P\, \varvec{x}_p^\mathrm{T}\, {\tilde{X}}\, \varvec{{\tilde{u}}} \\&- \frac{1}{2} u_P^2\, K_{PP} + \varvec{{\tilde{y}}}\varvec{{\tilde{u}}} + y_P\,u_P \end{aligned}$$

by

$$\begin{aligned} 0= & {} \partial _{\varvec{\tilde{u}}} {{\mathcal {W}}} = -{\tilde{K}} \varvec{{\tilde{u}}} - u_P\, {\tilde{X}}^\mathrm{T} \varvec{x}_P + \varvec{{\tilde{y}}}\ \rightarrow \\ \varvec{{\tilde{u}}}= & {} {\tilde{K}}^{-1}\, (\varvec{{\tilde{y}}} - u_P\, {\tilde{X}}^\mathrm{T}\, \varvec{x}_P) \end{aligned}$$

and

$$\begin{aligned}&0 = \partial _{u_P}W= - \varvec{x}_P^\mathrm{T}\,\tilde{X}\, \varvec{{\tilde{u}}} - K_{PP}\, u_P + y_P \\&0= y_P -{\varvec{x}}_p^\mathrm{T}\tilde{X}\, \tilde{K}^{-1}\varvec{\tilde{y}} -u_P(K_{PP} - \varvec{x}_P^\mathrm{T}\tilde{X} \tilde{K}^{-1}\tilde{X}^\mathrm{T}\varvec{x}_P) \end{aligned}$$

If one denotes the optimum loads of the previous $P-1$ inputs by $\varvec{{\tilde{u}}}' = {\tilde{K}}^{-1}\, \varvec{{\tilde{y}}}$, one can express the optimality conditions using $\varvec{x}_P^\mathrm{T}\, {\tilde{X}}\, \varvec{\tilde{u}}'= \varvec{x}_P^\mathrm{T}\, \varvec{w}$, as

7 $$\begin{aligned} u_P= & {} \frac{y_P - \varvec{x}_P^\mathrm{T}\, \varvec{w}}{K_{PP} - \varvec{x}_P^\mathrm{T}\, {\tilde{X}}\, {\tilde{K}}^{-1}\, {\tilde{X}}^\mathrm{T}\, \varvec{x}_P }\nonumber \\ \varvec{{\tilde{u}}}= & {} \varvec{{\tilde{u}}}' - u_P\, {\tilde{K}}^{-1}\, {\tilde{X}}^\mathrm{T}\, \varvec{x}_P\ \end{aligned}$$

The update rules for $\varvec{u}$ from Eq. (7) require computation of the inverse of ${\tilde{K}}$, which, a) is computationally costly and, b) biologically not straightforward. I therefore derived an iteration rule using the Sherman–Morrison–Woodbury identity (Nocedal and Wright 2006), which yields an iteration equation for $K^{-1}$ from the $P-1$st to the *P*th pattern

8 $$\begin{aligned} K^{-1} = \left( \begin{array}{cc}{\tilde{K}}^{-1} &{}\varvec{0}\\ \varvec{0}^T&{}0\end{array}\right) + {{\mathcal {C}}}_P^{-1} \left( \begin{array}{cc}\varvec{{\tilde{Q}}}\varvec{{\tilde{Q}}}^T &{}-\varvec{\tilde{Q}}\\ bm{{\tilde{Q}}}^T&{}1\end{array}\right) \end{aligned}$$

with $\varvec{{\tilde{Q}}}={\tilde{K}}^{-1}\,{\tilde{X}}^\mathrm{T}\, \varvec{x}_P$ and ${{\mathcal {C}}}_P=K_{PP} - \varvec{x}_P^\mathrm{T}\,{\tilde{X}}\, {\tilde{K}}^{-1}\, {\tilde{X}}^\mathrm{T}\, \varvec{x}_P$. The iteration equation (8) can be proven by elementary algebra ($K^{-1}\, K = 1\!\mathrm{l}$).

#### Remarks

- Translation of update rules from Eq. (7) to weight updates $\varvec{\Delta w} = X \varvec{\Delta u}$ is straightforward:
  $$\begin{aligned} \varvec{\Delta w}= & {} \left( {\tilde{X}}, \varvec{x}_P\right) \left( \begin{array}{cc}\varvec{{\tilde{u}}}-\varvec{\tilde{u}}'\\ u_p\end{array}\right) = (-{\tilde{X}}{\tilde{K}}^{-1}\tilde{X}^\mathrm{T} + 1\!\mathrm{l})\, \varvec{x}_P\, u_P\\= & {} (y_P - \varvec{\tilde{x}}_p^\mathrm{T}\, \varvec{w})\, \frac{(1\!\mathrm{l}-{\tilde{X}}\tilde{K}^{-1}{\tilde{X}}^\mathrm{T})\varvec{x}_P}{\varvec{x}_P^\mathrm{T}(1\!\mathrm{l}-{\tilde{X}}\tilde{K}^{-1}{\tilde{X}}^\mathrm{T})\varvec{x}_P}; \end{aligned}$$
  see result from Eq. (5).
- $1\!\mathrm{l}-{{\mathcal {N}}}:={\tilde{X}}\, {\tilde{K}}^{-1}\, {\tilde{X}}^\mathrm{T}$, and ${{\mathcal {N}}}$ are projection operators, since $[1\!\mathrm{l}-\mathcal{N}]^2=[1\!\mathrm{l}-{{\mathcal {N}}}]$ and ${{\mathcal {N}}}^2={{\mathcal {N}}}$.
- If $P-1\le N$ and patterns are linearly independent, ${\tilde{K}}$ is a Gramian and, hence, invertible.
- For $P-1$ exceeding *N*, ${\tilde{K}}$ can no longer be exactly inverted. Formally this is not necessary using a kernel representation, since the kernel operates on an infinite-dimensional Hilbert space. Biologically, for a finite number *N* of neurons, approximate inversion can be obtained by importance scaling (see below).
- Recursively adding data points continuously increases the dimensions of the matrix $K^{-1}$ and, hence, memory and computational costs. A brute force strategy to avoid this numerical divergence is to introduce a cutoff dimension, after which one removes the patterns with lowest importance values *a*. For all figures except Fig. 5, in which we explicitly study this parameter, we used a cutoff dimension of 300.

### Importance scaling

Importance is introduced by attenuation factors $0 \le a_t\le 1$ that scale the inequality constraints of support vector regression: $-(\varepsilon + \zeta _n) \le a_n\, [y_n - f(\varvec{x}_n)] \le \varepsilon + \zeta _n^*$. If $a_n$ is small, slack variables can also be small and the pair $(y_n,\varvec{x}_n)$ contributes little to the loss via the regularizer. The resulting optimal solution is very similar to the one without attenuation factors, only the weight vector are now

$$\begin{aligned} \varvec{w}=\sum _t u_t\, a_t\varvec{x}_t \end{aligned}$$

which, in the computation of the recursive learning rule, requires to replace

$$\begin{aligned} \kappa (n,m) \rightarrow \kappa (n,m)\, a_n\, a_m\ . \end{aligned}$$

Biologically, this rule maps to an attenuation of the inputs $\varvec{x}_t\, \rightarrow a_t\, \varvec{x}_t$. Thus, patterns with low $a_t$ are treated as more different to patterns with large $a_t$, even if they have similar structure.

The scaling of the kernel also has interesting consequences for situations in which the *K* is no longer invertible ($P>N$) if constructed from a finite population of neurons. In this case, one nevertheless, can apply the iteration equation (8); however, patterns with small $a_n$ will contribute only little to $\varvec{\tilde{Q}}$ as the respective rows are scaled down in ${\tilde{X}}^\mathrm{T}$. The resulting matrix is hence no longer an exact inverse, but the patterns for which the “inversion” fails mostly are those with low $a_n$. This is best illustrated by assuming $a_n=0$, in which case the pattern $\varvec{x}$ has no contribution to $\varvec{{\tilde{Q}}}$ and hence $K^{-1}$, as if it would not have been used for learning. Functionally modulating plasticity with *a* also allows a post hoc improvement in an existing episodic memory, by setting higher importance $a_n$ to this pattern if the episode is presented as second time.

### Theta sequences

Sparse binary random patterns $\varvec{\xi }_n$ with Prob($\xi _n^{(k)} = 1$) $=f$ are assumed to represent hippocampal ensembles that fire together at a specific phase of the theta cycle. Given that *S* of those ensembles are activated in sequence during a theta cycle the population pattern in cycle *t* equals

$$\begin{aligned} \varvec{x}_t = \sum _{k=0}^{S-1} \varvec{\xi }_{t+k} \end{aligned}$$

For a population of *N* neurons, the overlap of two such patterns can be computed as

$$\begin{aligned} K_{nm}= & {} \sum _{kk'} \varvec{\xi }_{n+k}^\mathrm{T}\varvec{\xi }_{m+k'} {\underset{N\rightarrow \infty }{\rightarrow }} [S-\vert n-m\vert ]^+ \langle \xi ^2\rangle \, N \\&+ (S^2-[S-\vert n-m\vert ]^+) \langle \xi \rangle ^2\, N\ . \end{aligned}$$

For independent binary random variables, one finds $\langle \xi \rangle = \langle \xi ^2 \rangle = f$, and thus the overlap is a linear triangular kernel

$$\begin{aligned} K_{nm}= & {} K(\vert n-m\vert )\\= & {} N\, \left( [S-\vert n-m\vert ]^+\, f\, (1-f) + (Sf)^2 \right) \ \end{aligned}$$

as depicted in Fig. 2.

### Frequency kernels

The cochlea separates a sound *s*(*t*) into frequency channels that roughly act as band-pass filters and can thus be characterized by a filter kernel $\gamma _f(t)$, with *f* denoting the center frequency of the cochlear channel. If one assumes multiple ($k=1,\ldots ,K$) auditory nerve fibers to connect to such a frequency channel the linear response of each of those fibers can be modeled as $x^{(k)}_t = c_f(t-\Delta ^{(k)}) = (\gamma _f *s)(t-\Delta ^{(k)})$ with a fiber-specific delay $\Delta ^{(k)}$ that may reflect differences in fiber lengths, diameters or myelination.

For a large number *K* of fibers the resulting kernel can be computed as an integral

$$\begin{aligned}&{\sum _k x^{(k)}_t\, x^{(k)}_{t'} \approx \int \mathrm{d}\Delta \, c_f(t-\Delta )\, c_f(t'-\Delta )} \\&\quad = {\int \mathrm{d}u\, c_f(u)\, c_f(t'-t+u) = \kappa (t'-t)}\ , \end{aligned}$$

which corresponds to the autocorrelation of cochlear response, and for long broadband signals *s* equals the autocorrelations of the filters $\gamma _f$. The exponentially decaying kernel used in Fig. 6 reflects exactly such a prototypical autocorrelation.

Specifically, a sound signal (the beginning of the CC BY NC song *I’ll be your everything* by Texas Radio Fish, http://ccmixter.org/files/texasradiofish/63300) was passed through a gamma tone filterbank consisting of seven channels (center frequencies $2^k\times 200$ Hz, $k=0,\ldots ,6$) with width constants $2.019 ERB$ (Glasberg and Moore 1990). In each of the channels $\rho _k$ data points per cycle (equally spaced) were selected for learning. The parameters $\rho _k$ where channel (*k*-)dependent and equaled 6, 4, 3, 3, 1.5, 1, .25 for $k=0,\ldots ,6$. The recursive KSVR was fitted in each channel independently in chunks of 500 data points.

For full audio reconstruction, the reconstructed signals were Fourier-transformed in each band and divided by the Fourier transforms of the respective gammatone filter kernel omitting frequencies below 10 Hz and above 20 kHz. These filter-corrected components were backtransformed, summed and rescaled to the root mean square level of the original signal.

## Discussion

Kernel support vector regression (KSVR) is a powerful tool for function fitting. Here, I presented a biologically plausible neural implementation of recursive KSVR that enables storing episodic memories as temporal sequences of retrieved sensory-motor activity patterns $y_t$ (i.e., fitting $y_t$). The kernels can be biologically interpreted as scalar products of activity patterns $\varvec{x}_t$ of a reservoir and provide a neural representation of temporal distance.

Hippocampal theta sequences provide a well-known example that realize exactly such a reservoir. However, already in the hippocampus, neuronal activity not only consists of sequence-type activity, but also exhibits rate modulations induced by changes in the sensory environment generally known as remapping (Muller and Kubie 1987; Leutgeb et al 2005; Fetterhoff et al 2021). Thus, behavior-related neuronal activity may always contain both reflections $W\varvec{x}_t$ of the reservoir and feedforward sensory motor drive, thereby balancing expectations (i.e., reservoir-driven activity) and sensory reality. This combination of top-down and bottom-up input streams is widely considered to be a general design principle of the neocortex (Douglas and Martin 2004; Larkum 2013), resulting in sensory-motor activity patterns $y_t$ at the same time reflecting stimulus-driven responses and intrinsic dynamics as, for example, reflected by synfire chains (Abeles et al 1993).

While the view of neocortex as a hierarchical combination of sensory-motor prediction loops (Ahissar and Kleinfeld 2003) is probably a good proxy of the neurobiological substrate, it is not widely explored in classical artificial neural network research. There, the universal approximation theorem, as a hallmark result, states that neural networks can approximate any function to arbitrary degree of precision (Cybenko 1989; Hornik 1991) which rather views brains as feedforward function fitting devices. The field of reservoir computing has extended this idea toward the temporal domain by suggesting intrinsic neural dynamics to represent a time axis as the independent variable of function fitting (Jaeger 2005) and thereby allows neural networks to generate predictions varying with time. However, to be able to operate on a continuous stream of sensory inputs, the learning rules for the output synapses of the reservoir need to be able to recursively update (Williams and Zipser 1989; Stanley 2001; Sussillo and Abbott 2009), which requires a biological interpretation of the common least-mean square derived ideas.

Here, I suggest that the iterative update of the projection operation ${{\mathcal {N}}}$ that only requires anti-Hebbian type outer products can be implemented as anti-Hebbian learning of a simple recurrent neuronal network: In the neural space of synaptic weights, $X\, K^{-1}\, X^\mathrm{T} = 1\!\mathrm{l}-{{\mathcal {N}}}$ is of outer product form as seen from Eq. (8). The matrix ${{\mathcal {N}}} = 1\!\mathrm{l}- X\, K^{-1}\, X^\mathrm{T}$ can thus be interpreted as the connectivity of a recurrent neural network that is learned by anti-Hebbian updates, i.e.,

9 $$\begin{aligned} {{\mathcal {N}}} = 1\!\mathrm{l}- \sum _{t=1}^{P-1} \varvec{r}_t\, \varvec{r}_t^\mathrm{T}\ \end{aligned}$$

with $\varvec{r}_P= {{\mathcal {N}}}\varvec{x}_P/\sqrt{\varvec{x}_P^T{{\mathcal {N}}}\varvec{x}_P}$. Since $X\,K^{-1}\, X^\mathrm{T}$ is a projection matrix (see Sect. 3), one furthermore can write $\varvec{r}_t=\varvec{x}_t^{\perp }/\Vert \varvec{x}_t^\perp \Vert$ with $\varvec{x}^\perp _t$ being the component of $\varvec{x}_t$ that is orthogonal to all previously learned patterns.

This leads to the following interpretation of $\varvec{r}$ as the activity of a neural network in discrete time *s*

$$\begin{aligned} {\varvec{r}}(s+1) = \phi [\delta _{s,0}\,{\varvec{x}} + {{\mathcal {N}}}\varvec{r}(s)]\ , \end{aligned}$$

where the network is initialized at $\varvec{r}(s=0)=0$, the input $\varvec{x}$ is present only at time step $s=0$, and $\phi (\varvec{z})=\varvec{z}/\Vert \varvec{z}\Vert$. As a result of this dynamics $\varvec{r}(s)=\varvec{x}_t^{\perp }/\Vert \varvec{x}_t^\perp \Vert$ for all time steps $s>1$. This dynamical fixed point state will then produce an anti-Hebbian weight update from Eq. (9).

A further drawback of RLS-derived rules was their lacking theoretical foundation since they made explicit use of the reservoir patterns that, for technical reasons, were limited to a small subsample of neurons. Here, I use the generalized representer theorem (Schölkopf et al 2001) to translate the weight update into an update rule for the loads (coefficients) $\varvec{u}$ of the input patterns *X* and thereby avoid an explicit representation of the neural feature space $\varvec{x}_t$ and instead only require a kernel representation (Hermans and Schrauwen 2012). Formulation of the learning rule on the loads allows analytical insights for reservoirs of size $N\rightarrow \infty$, but also reduces the computational demand of simulating (or recording) from a large number of neurons.

Importantly, this paper considers reservoir activity only in the context of memory retrieval but not replay of reservoir sequences. Replay in the context of reservoirs has often been used to improve performance and stability (Mayer and Browne 2004; Jaeger 2010; Sussillo and Abbott 2012; Reinhart and Jakob Steil 2012; Laje and Buonomano 2013; Jaeger 2017; Leibold 2020). However, changing reservoir patterns would require to also change the readout-matrix to maintain the originally learned memory traces $y_t$ (Sussillo and Abbott 2012; Reinhart and Jakob Steil 2012; Jaeger 2017). In the context of the model presented here, relearning is not necessary as long as the kernel remains fixed, i.e., the topology of the space is constant. Neurobiologically, however, such a trick would require to change the weights $\varvec{w}$ by replacing the matrix *X*.

I presented two neurobiological examples of how kernel representations are or may be implemented, hippocampal theta sequences and auditory nerve fiber populations. Temporal sequences of activation patterns, however, are ubiquitous in sensory-motor systems and occur on multiple time scales. Thus the proposed theory may also apply to a multitude of other examples. A prerequisite is to find a continuous representation of time in the population patterns that then translates via a scalar product into kernels with continuous time dependence. Further such examples could be the long-term changes of the hippocampal rate code of place cells (Mankin et al 2012; Ziv et al 2013), activation of cerebellar purkinje cells during limb movements (Hewitt et al 2011), or olfactory-driven activity that evolves along fixed trajectories after odor presentation (Stopfer and Laurent 1999).

## Supplementary Information

Below is the link to the electronic supplementary material.

Original movie snippet from *.re_potemkin*, a copyleft crowdsourcing free/open source cinema project (https://re-potemkin.httpdot.net/)

Reconstruction of the original movie snippet (Video 1).

Original movie with time extended in the middle.

As Supplemental Video 3 but with a new scene added post hoc.

Reconstruction of original movie (Video 1) with importance reduced in the last frames.

Reconstruction of original movie (Video 1) with importance reduced in the first frames.

Filtered version (see Methods) of the original sound snippet song from the song *I{\rsquo}ll be your everything* by Texas Radio Fish (http://ccmixter.org/files/texasradiofish/63300, CC BY NC)

Reconstruction of Supplemental Audio 1.

## Data Availability

The core recursive KSVR implementation can be found at https://github.com/cleibold/recsvr.
